# Exploring the perceived challenges and support needs of Indonesian mental health stakeholders: a qualitative study

**DOI:** 10.1186/s13033-021-00504-9

**Published:** 2021-11-08

**Authors:** Adelia Khrisna Putri, Nuvi Gustriawanto, Satwika Rahapsari, Anna Rusdiyana Sholikhah, Sanidya Prabaswara, Annisa Cahya Kusumawardhani, Susi Ari Kristina

**Affiliations:** 1 Faculty of Psychology, Universitas Gadjah Mada, Jl.Sosio Humaniora, Yogyakarta, 55281 Indonesia; 2 Faculty of Pharmacy, Universitas Gadjah Mada, Jl.Sekip Utara, Yogyakarta, 55281 Indonesia

**Keywords:** Mental health services, Support needs, Perceived challenges, Qualitative research, Indonesia

## Abstract

**Background:**

Despite the large treatment gap in Indonesia, limited studies have attempted to explore both service users’ and providers’ evaluations of the current mental health system holistically. This study aims to explore the perceived challenges and support needs of Indonesian mental health stakeholders.

**Methods:**

This qualitative study collected data from 17 participants from two mental health stakeholders in Yogyakarta (i.e., health professionals and service users) through a semi-structured interview. Thematic analysis was used to analyze the data.

**Results:**

Findings reveal that service providers and users shared equally strong concerns regarding challenges and needs for improving mental health literacy, accessibility to services, and government support. However, a distinct emphasis was made in several areas—with service providers hinting more towards issues with interprofessional collaboration. In contrast, service users emphasized the negative attitude of health professionals and poor accessibility to service information.

**Conclusion:**

The mental health service system is challenged by the lack of accessibility to service information, the limited spread of mental health practitioners, stigma, and lack of mental health literacy among both the public and professionals. A need for improvement in mental health promotion, accessibility, and quality of mental health workers is highlighted to satisfy the needs of both service users and providers.

## Introduction

There is a growing public concern about improving the care for people with mental health problems and minimizing the treatment gap. In Low and Middle-Income Countries (LMIC), treatment gaps have been estimated to be above 90%, even reaching 95% in rural Indonesia [1]. The combination of limited mental health services [2], scarce mental healthcare professionals [3], and mental health stigma [4] are among some reasons that have contributed to this gap despite the high number of people who experience mental health problems. In Indonesia, the prevalence rates in households with family members who have schizophrenia are 6.7% or 282,654 households from a total of 218,716 sample households [5]. Meanwhile, the prevalence of depression and other mental-emotional disorders reaches 5.9%. Unfortunately, the report shows that only 9% of patients with depression receive treatment, and 48.9% of schizophrenia patients receive routine medication [5].

The health services in Indonesia are decentralized and devolved upon provincial and district governments under the Ministry of Home Affairs. The local governments are responsible for planning and managing the health system, including the mental health system. In 2014, The House of Representatives approved Indonesia’s mental health law which mandates every province to have at least one mental health hospital [6]. The availability of mental health services is expected to reduce traditional treatments of mental disorders commonly used by some communities. However, seven provinces in Indonesia do not have a mental hospital [2], highlighting that access to mental health services is not yet evenly distributed.

Another major issue was the lack of mental health literacy [7]. In the past, poor help-seeking behavior is often perceived as a great contributor to the high treatment gap [8]. Two major driving forces that prevented seeking help were stigma [9] and lack of mental health literacy [10]. People with mental disorders in Indonesia still carry a stigma which, in some cases, leads to physical restraint and confinement (*pasung*) by the family [4]. It also led them to seek help from spiritual or religious leaders for treatment. In other words, the treatment gap has primarily been attributed to the patient’s lack of initiative and resolve to seek the available treatments. Nevertheless, some people reported that their dissatisfaction with existing health services primarily caused their hesitation in seeking treatment [11]. A recent Indonesian study highlighted that help providers (i.e., faculty members) also face real challenges in providing aid for students in distressed due to paucity of mental health services, lack of accessibility to information, and complicated professional boundaries [12]. Therefore, strategies to improve the quality of mental health services should consider the challenges experienced by both service users and health professionals.

This study was conducted in Yogyakarta, which is one of Indonesia’s 34 provinces. Yogyakarta is a city situated on central Java Island. It is one of the most densely populated provinces of Indonesia. Surveys have shown that Yogyakarta ranks first in public knowledge about accessing health services in Indonesia [5]. In Yogyakarta, mental health services are more easily accessible to the community at various places (i.e., hospitals, consulting bureaus, and primary public healthcare). In 2004, the Sleman district in Yogyakarta, in collaboration with the Faculty of Psychology Universitas Gadjah Mada, conducted a pilot project of integrating psychologists into primary health care in Indonesia [13]. The project resulted in placing psychologist in every primary healthcare, especially in Sleman, Bantul, and the city of Yogyakarta.

In this study, mental health service users and practitioners were interviewed in-depth to elicit their views on priorities for evaluating and developing support services for people with common mental health problems. More specifically, we focused on evaluating unmet needs, challenges in seeking, receiving, and providing sufficient support, and the expected improvements.

## Methods

### Study design and participants

This study is an explorative qualitative study of mental health stakeholders. There are two main groups of participants: health professionals and service users. Being an active health professional in an Indonesian health service center was the only inclusion criterion for the health professional participants. The inclusion criteria for service users were (a) Have a patient health questionnaire (PHQ-9) score above > 9 or have been clinically diagnosed with depression or anxiety by a psychologist/psychiatrist; (b) Have previously accessed a health service center for their mental health issue, (c) Speaks Indonesian.

To collect data on various perspectives regarding mental health services, we employed two strategies. We mainly used the snowball sampling technique for the health professionals, whereby we invited one health professional from a public hospital who then proceeded to recommend several other health professionals. However, this sampling strategy could not be fully implemented because (1) Most health professionals could only recommend colleagues who work in the same field, limiting the diversity in the sample of health professionals; (2) Some of the recommended experts were not available for interview. As such, the sampling strategy was modified. The researchers contacted a private counseling center and one public health center to gain the views of health professionals who work in a non-hospital setting. In the end, as many as three psychologists, three psychiatrists, two general practitioners, and one clinical pharmacist participated in this study in 2019.

Service users were recruited through an online survey link shared through various social media platforms (WhatsApp, Facebook, & Instagram). The survey has two parts: (1) socio-demographic questions and (2) close-ended questions to measure clinical condition (i.e., PHQ-9) and experience in help-seeking behavior. At the end of the survey, participants were given the choice of providing a phone number if they agree to be contacted further for an interview. Of 95 service users who completed the survey, only 25 agreed to be contacted for an interview. After eliminating those who did not meet the inclusion criteria, eight service users were interviewed. We did not add more participants (n = 17) because data saturation was reached during the analysis. Regular comparisons were made between analyses to determine the frequency of new themes emerging—data collection stops once no new information can be gathered [14]. After analyzing the 6th participant in each group, very minimal new information was gained, prompting us to continue data collection for 2–3 more interviews in each group to confirm that no other themes appeared.

### Procedure

Interview guidelines, information sheets, informed consent, as well as the online survey were designed by the research team. To promote patient-public involvement (PPI), the research team also involved a mental health user, who advised on the wording and the domains to cover during the drafting of the interview guideline and who was also involved throughout the data collection and analysis process. Next, participants were collected using the sampling strategies outlined in “Study design and participants” section.

Before the interview session, participants were given an information sheet explaining the study’s objectives, participant criteria, research team, and the confidentiality agreement. All participants have signed informed consent before data collection. Two researchers for each interviewee conducted an hour-long interview which were audiotaped and transcribed. Each interview was structured around a set of central questions. For service users, the questions revolved around the subjects’ evaluation of currently available support and unmet needs. Questions for the health professionals were directed on the challenges they faced in providing treatment and the support they needed to improve health care. Each question was followed by several probe questions to fill in more details. In general, the service users were asked two key questions:

- What are some of the issues with current mental health services?
- What services are missing from the current range of services provided as support for people with common mental disorders in Indonesia?
  Similarly, there were questions for health professionals:
- What are some of the challenges in treating people with a common mental disorder?
- What support would you need to improve on the mental health service in Indonesia?

### Analytic approach

The data were analyzed using an inductive thematic analysis approach [15]. All the interview transcripts were imported to Nvivo12 as the platform used to organize and code the data. First, all authors familiarized themselves with the data while taking notes of initial coding, which were then discussed with the team to decide on essential sub-themes. Two of the authors then coded the entire dataset based on the agreed sub-themes. Data from the health professionals and service users were still analyzed separately at this point. Afterward, themes from each group were compared to look for commonalities and differences and later narrowed down into overarching categories by the entire team. Any disagreements surrounding categorizations were discussed and resolved as a team. Data analysis was conducted in Indonesian to prevent the potential loss of meaning through translation. The transcripts were translated into English when the analysis process moved to map the interpretations.

## Results

### Socio-demographic description

Of the 17 participants interviewed in this study, 9 were professional health providers, and 8 were service users. All service users have experienced treatment from a psychologist, ranging from trying 1–6 different psychologists. Two participants have also contacted a psychiatrist. Although not all participants provided information of where they accessed this help, some have stated private practices, hospitals, community health centers, and university health centers as their primary contact. Detailed information of the participants is summarized in Table 1.

**Table 1:** Socio-demographic summary of participants

Characteristics	Stakeholder groups		
	Mental health workers (N = 6)	Health workers (N = 3)	Service users (N = 8)
Gender			
Female	5	2	7
Male	1	1	1
Age group			
20–25			5
26–30	1	1	2
31–35	1		1
36–40	1	1	
41–45		1	
46–50	3		
Highest level of education			
Senior high school			3
Undergraduate		3	2
Postgraduate	6		3
Employment status			
Employed	6	3	4
Self-employed			4
Length of employment (years)			
2–5		1	
> 5	6	2	

*N* number of participants in each group

### Emerging themes

Generally, both service users and health practitioners provided similar insight into the challenges within the current mental health system. However, some challenges were more uniquely stressed by health practitioners and service users, respectively (see Table 2).

**Table 2:** Descriptive themes

Major theme	Sub-theme	Issue raised by^a^	
		Health professionals (N = 9)	Service users (N = 8)
Social challenges	Lack of mental health literacy	9	7
	Negative attitude of health professionals	4	7
Structural challenges	Limited accessibility to services	6	7
	Unintegrated mental health management	6	4
Bridging the care provision gap	Mental health as a major government agenda	7	6
	Diversification of mental health promotion and education	6	8
	Interprofessional collaboration practice	8	4

*N* number of participants in each group

^a^The number of participants that raised the theme

### Social challenges

#### Lack of mental health literacy

Most participants identified lack of mental health literacy as one of the leading social challenges when dealing with mental health services, particularly stigma. Family stigma was described as a significant barrier to help-seeking behavior and treatment adherence. Service users claimed that negative family communication style—which was often judgmental—could burgeon into a form of internalized self-stigma, decreasing their motivation to seek or continue treatment. A service user stated:

Umm … most said that I was distant from God, lacked faith, or seemed like an overly sensitive person … that came from my own family. It was like I became depressed because I exhausted myself from wanting to achieve too many things … At one point, I started to think, do I really need medication for simple stress?

Health professionals also raised the issue of poor mental health literacy interfering with treatment adherence. They added that most patients expected treatments or psychiatric medications to work instantly like a medication for physical illness. A clinical pharmacist stated, “They tend to stop drinking the medicine once they feel better. This ultimately leads to a form of relapse ….”

A health practitioner explained that some families also hid family members who suffered from mental health and went to the extent of deleting them from the family registry, further preventing individuals from receiving care and health insurance.

#### Negative attitude of health practitioners

Another sub-theme relating to stigma was the attitude of mental health workers toward patients. Several service users reported feeling devalued and dismissed by some health professionals, which led them to delay or stop treatment for several years. A few service users emphasized that this attitude became apparent mainly when presenting religious or sexual orientation cases. This claim suggests that the open-mindedness of the health workers toward sensitive issues (i.e., culture and religion) must be perceptible to the patients.

I’ve stopped coming to mental health practitioners for now. I feel like my case can’t be solved here because it was clear to me—by how they reacted to my story—that mental health practitioners were still very sensitive toward such issues as atheism and sexual orientation … So, I don’t believe that mental health workers are ready to treat such unique cases (service user).

Another issue raised by service users was the perceived unresponsiveness of health practitioners. This problem was raised by several service users who mainly observed this within the context of health practitioners in training. Several service users explained,

A professional-in-training handled my case. I understand that sometimes students would be given a chance to treat us while supervised by a senior practitioner. However, I came here to receive professional treatment from a certified and competent person … We (service users) know when we’re handled by a professional or a trainee. At the very least, we should still be given more sessions with a certified psychologist.”

These responses show that some service users are dissatisfied with having trainees at the front line of the service delivery, pointing towards the need for mental health services to re-evaluate the training given to trainees before they are incorporated in treating patients.

### Structural challenges

#### Lack of accessibility to service

One of the conspicuous gaps in the service provision was the accessibility of mental health services. Service users pointed out that mental health professionals are scarce and that the spread is relatively uneven—located mainly in major cities. Also, it is not easy to find information about mental health services. A service user commented,

Before we talk about the quality of the services, I feel more effort needs to be put into ensuring that people know these services even exist. Not everyone is privileged enough to access it. Even though the government has provided national health insurances, the fact remains that many people are still unaware that such services are available.

Real challenges were also noted for service users who live farther away from the city center. Distance hinders service accessibility by adding to the financial burden of the patients.

#### Unintegrated mental health management

Another issue strongly stressed, particularly by health professionals, was the poor referral system among health practitioners (doctors, psychiatrists, psychologists). They complained that the various branches of medicine in the health system were still not integrated and confined within their independent professional bodies, resulting in poor interprofessional communication. Although professional teamwork between doctors and psychiatrists is becoming increasingly common, teamwork between medical and non-medical practitioners remains limited. Service users added that the lack of a referral system between practitioners sometimes contributed to treatment delays because patients needed to be reassessed whenever they switched practitioners. Such an experience could also lower patients’ motivation for continuing treatment.

### Bridging the care provision gap

#### Mental health as a major government agenda

Service users highlighted that most people who seek help, particularly young people, often find it through online services. Despite being financially independent of the government, these online services acts as a bridge between the people and mental health services, providing a secure and accessible form of mental health support. The need for the government to formalize some of these initiatives by paying fair wages to service providers to sustain them is highlighted by a service user.

There are no online services such as suicide hotline from the government, only those made by youth. Even those (services) are starting to shut down because they’re receiving too many clients … with little support, because it is voluntary based … Meanwhile, those services help encourage people to seek professional treatment. If these services aren’t available, people won’t know where to go and whom to ask for treatment.

Both service users and health practitioners have stressed that the government should consider allocating more financial support for primary health centers. The currently limited government funding has brought little financial security for those involved, despite significant work demands. As a result, healthcare providers are not paid well, which could affect their commitment to improving the way they treat their patients. Several respondents felt that the government pays more attention to physical health problems, reflected in the small number of government policies implemented for mental health. The participants urged for mental health to be given higher priority in the government’s agenda.

#### Diversification of mental health promotion

Two specific means were highlighted for the promotion of mental health services: online and offline promotion. Most respondents agreed that current mental health promotion is correct in using social media to reach the younger population. Providing accessible online services (online counseling, support groups) and mental health information could help increase mental health awareness and attract more people to access service information.

Offline promotions are equally highlighted as a necessary means to reach the older population and those with limited access to the internet. Several strategies were suggested i.e., (1) Making mental health leaflets more available at public places, (2) increasing community psychoeducation, (3) integrating mental health knowledge in early education, (4) providing regular mental health screening for at-risk populations. Some suggestions have been made by the government—albeit not at all districts. For instance, only a few districts have organized early mental health screening programs on school grounds despite the benefits being gained.

#### Interprofessional collaboration practice

One of the themes strongly highlighted by participants across health professions was the poor interprofessional collaboration that persisted among them. As previously explained, the referral system between mental health and medical practitioners still lacked, partly due to the stigma and competition between practitioners of the two therapies. The competition was evident even to the service users.

There should be interdisciplinary teamwork between professionals… sometimes, there is still a sense of competition between psychiatrists and psychologists. There should be a clearly defined boundary that shows which patients should go to psychologists, psychiatrists, or medical doctors.

Although several respondents highlighted the strains in interprofessional relations, nearly all health professionals admitted the importance of strengthening the communication between professionals in various streams. There was a need to impart basic knowledge about other fields to improve the quality of service. For instance, some general practitioners needed basic training in assessing mental health problems because patients mainly came with physical complaints, making it difficult to distinguish those who exhibited mental health problems.

Strategies to improve interprofessional collaboration practices must start by incorporating them into the teaching curriculum for professionals-in-training. Trainees must be given every opportunity to familiarize themselves with various physical settings and how to communicate with other health professionals. Several suggestions to improve the mental health training curriculum were made—

… we (psychologists) deal with depressed patients with chronic or, sometimes, degenerative illnesses. So, the comorbidity with physical illness is high, adding to its complexity … It is essential and urgent for psychologists in training to also have placements in general hospitals to visit patients from the non-mental health-related ward (e.g., maternity ward, intensive care unit). So far, we’re mostly limited to the psychiatric ward.

Additionally, several service users also highlighted the need to re-evaluate the curricula to improve the practitioner’s diversity of skills. It is advised that training curricula expose mental health professionals to more culturally sensitive issues often considered taboo in the community. Lack of diversity of skills could result in health professionals showing a negative attitude toward culturally sensitive cases.

### Comparison between service providers and users

**Fig. 1:**
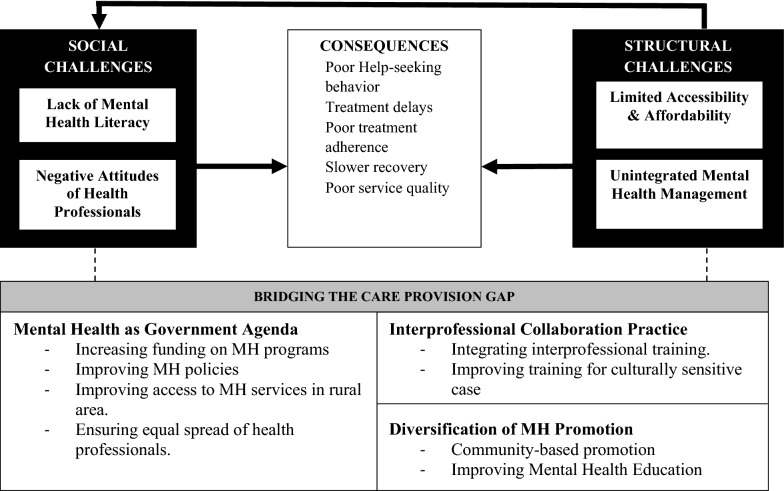
Schematic diagram of expressed challenges and needs of mental health stakeholders. The bold arrow reflects the perceived relationship between themes as described by participants. The dotted line reflects the improvement strategies that participants suggested to bridge unmet needs

Overall, service providers and users share equally strong concerns regarding improving mental health literacy, accessibility to services, and government support to maintain quality care. However, a distinct emphasis was voiced in some areas—with service providers strongly highlighting issues on interprofessional collaborations while service users were stressing more about service users’ attitudes and access to service information. These in-depth interviews resulted in critical solutions for improving mental health services for people with mental disorders: Fig. 1 presents the perceived challenges and needs mentioned by the mental health stakeholders.

## Discussion

The current findings complement several other past studies. The limited professional communication among health practitioners (doctors, psychologists, psychiatrists) was noted in a recent qualitative study of clinicians. Communication barriers within the emergency department were often found among practitioners in various disciplines [16]. Various challenges surrounding the lack of information sharing between health professionals were highlighted in studies in Japan [17] and the United States [18]. Despite the challenges in implementing interprofessional collaboration practice (IPC), the latter has generally been acknowledged to positively influence the quality of patient care [19] and work satisfaction and motivation among professionals [20], as also a reduction in the healthcare cost [21]. The above studies reinforce the respondents’ suggestions that IPC is adequately implemented.

The suggestion of some health providers and service users to re-evaluate and incorporate IPC into the training curricula for future health providers is also justified. Some medical schools have changed their curricula to foster more interprofessional collaboration skills through various experiential learning opportunities e.g., interprofessional workshop programs [22] and digital case studies [23]. These changes aim to familiarize university students with communicating and working with other health practitioners and addressing clinical cases in other health fields that one’s expertise could influence. However, families and communities involvement must also be equally strengthened.

In most LMIC such as Indonesia, patients’ family, and community tend to play a critical role in making treatment decisions (e.g., when and from where to seek help, and the kind of support that would be provided at home) and the recovery of patients with mental illness. The deeply rooted culture of “caregiving” in most Asian countries caused many patients with mental disorders to live with their family caregivers [24]. However, the recovery outcomes in these cases vary significantly influenced by the family’s knowledge about mental health. As some of the respondents said, the family and community’s stigma attached to mental illness could decrease the family’s motivation to seek help. The lack of faith in the available support and low perception of need as barriers for help-seeking behavior is also in line with a study of bereaved young adults in the United Kingdom [25]. It is equally important to highlight that, whereas health professionals stressed the lack of mental health awareness of the patients and poor interprofessional collaboration, the service users in this study stressed the negative attitude of health professionals as a major challenge.

Indonesia’s strong cultural values and religious attitudes could partly explain some of the more specific types of stigmas experienced by service users. In line with the current findings, stigma plays a critical role in shaping the negative attitude of health professionals [26]. However, the current findings noted that health professionals’ negative attitude does not necessarily reflect stigma toward mental illness but specific culturally sensitive cases (e.g., sexual orientation, atheism). Historically, evidence suggests that health providers have had problems providing treatments for those within this sexual minority group [27]. Even though there are greater acceptance of sexual variations in more liberal countries in recent years, much less is evident in more religiously salient [28]. Hence, in countries such as Indonesia, some patients who expressed atheism could receive negative attitudes from their community. This situation points to the need to explore other ways of improving mental health services for minority groups in Indonesia and the cultural competence of health workers.

Lastly, a recent review of the mental health provision of LMIC has highlighted two main challenges similar to the structural issues found in this study: (1) lack of legislation and policies to direct mental health programs, and (2) lack of health budget allocation for mental health services [29]. Participants’ stress on the prioritization of mental health by the government and increasing budget allocation for improving mental health services accessibility and quality is justified.

## Strengths, limitation, and direction for future studies

The current study will inform the implementation of a key objective of the health strategy to improve mental health services for all. Most past studies focused primarily on evaluating barriers to help-seeking. However, not many attempted to include the patients’ views on how services could be improved. Even scarcer are studies that attempt to look holistically at both the service users’ and practitioners’ unmet needs as indicators of the ways to improve mental health care—a research gap answered by this current study. This study, therefore, provides insight into the challenges that were not shared equally by the various groups. This information could aid in improving the strategies used to address each challenge. The views were expressed by health professionals from various backgrounds, providing a more holistic perspective of the challenges faced by each profession, both in a hospital setting and at community-based healthcare centers. The current research team is comprised of health professionals, academicians, and a service user. This PPI ensured that views of both the health practitioners and service users were equally represented, starting from research formulation to the final categorization process [30]. Additionally, the data was collected exactly the year before the COVID-19 pandemic, providing valuable insight regarding the baseline challenges felt by participants, which future studies and policymakers could use as a comparison.

The study has several limitations. Firstly, our sampling method has resulted in an over-representation of service users who are financially stable, living in cities, and hold an undergraduate degree or above. Thus, the findings from this study may not reflect the unmet support needs of the rest of the population. Future studies should include perspectives from people from a lower socio-economic background and living in rural areas. As previous studies suggest, low income is one of the barriers to receiving mental healthcare services, and financial distress and stress might impact mental health [31]. Next, this study has not incorporated the views of traditional healers (shamans and alternative therapists) and religious leaders (priests and imams). The popular reasons attributed to mental illness pointed out by Human Rights Watch [32], which were also highlighted by one of our service user participants, are a widespread belief that mental illnesses are caused by evil spirits taking possession of a person because they have sinned, behaved indecently, or lacked faith in religion. Such beliefs are typically found more in Asian countries compared to Western countries. As a result, some families—particularly those living in rural areas—often consult traditional or faith healers that are more easily accessible [32].

## Conclusion

Overall, findings reveal that service providers and users share equally strong concerns regarding improving mental health literacy, accessibility to services, and government support to maintain quality care. However, some distinct emphasis was made in several areas. There were service users’ questions regarding the quality of mental health care received after the initial contact with the health professional. The need for better training for professionals and the lay public on how to respond to support needs is indicated, as is the need for enforcing interprofessional collaboration to improve patient care. Addressing the unmet needs described in this study would help improve the quantity and quality of mental health care and trust between mental health stakeholders.

## Data Availability

The datasets generated and/or analyzed during the current study are not publicly available due to the risk of identifying participants; but are available from the corresponding author on reasonable request.

## Abbreviations

IPC: Interprofessional collaboration practice
LMIC: Low and Middle-Income Countries
PHQ-9: Patient Health Questionnaire-9
PPI: Patient-public involvement
